# Synergistic Antifungal, Allelopatic and Anti-Proliferative Potential of *Salvia officinalis* L., and *Thymus vulgaris* L. Essential Oils

**DOI:** 10.3390/molecules23010185

**Published:** 2018-01-16

**Authors:** Ersilia Alexa, Renata Maria Sumalan, Corina Danciu, Diana Obistioiu, Monica Negrea, Mariana-Atena Poiana, Cristian Rus, Isidora Radulov, Georgeta Pop, Cristina Dehelean

**Affiliations:** 1 Faculty of Food Processing Techology, Banat’s University of Agricultural Sciences and Veterinary Medicine “King Michael I of Romania” from Timisoara, Calea Aradului, No. 119, Timisoara 300645, Romania; alexa.ersilia@yahoo.ro (E.A.); negrea_monica2000@yahoo.com (M.N.); atenapoiana@yahoo.com (M.-A.P.); ruscristian181@yahoo.com (C.R.); 2 Faculty of Horticulture and Forestry, Banat’s University of Agricultural Sciences and Veterinary Medicine “King Michael I of Romania” from Timisoara, Calea Aradului, No. 119, Timisoara 300645, Romania; 3 Faculty of Pharmacy, “Victor Babes“ University of Medicine and Pharmacy Timisoara, Eftimie Murgu Square, No. 2, Timisoara 300041, Romania; corina.danciu@umft.ro (C.D.); cadehelean@umft.ro (C.D.); 4 Interdisciplinary Research Platform, Banat’s University of Agricultural Sciences and Veterinary Medicine “King Michael I of Romania” from Timisoara, Calea Aradului, No. 119, Timisoara 300645, Romania; diana.obistioiu@yahoo.com; 5 Faculty of Agriculture, Banat’s University of Agricultural Sciences and Veterinary Medicine “King Michael I of Romania” from Timisoara, Calea Aradului, No. 119, Timisoara 300645, Romania; isidoraradulov@yahoo.com (I.R.); getapop_tm@yahoo.com (G.P.)

**Keywords:** *Salvia officinalis*, *Thymus vulgaris*, *Fusarium graminearum*, A375 human melanoma, B164A5 mouse melanoma

## Abstract

The current study aimed to investigate the chemical composition and the synergistic potential of two essential oils (EOs), as obtained from *Salvia officinalis* L. (SEO), and *Thymus vulgaris* L. (TEO). The antifungal potential was tested in vitro against *Fusarium graminearum* (Fg 06_17), the herbicidal effect was studied using weed seeds of *Amaranthus retroflexus* (ARET), *Chenopodium album* (CALB), *Echinochloa crus-galli* (EGAL), but also wheat seeds (WS) of the Lovrin variety and tomato seeds Saint-Pierre of the variety. The GC-MS profile highlights that the mains compounds identified in SEO were: caryophyllene (25.364%), camphene (14.139%), eucalyptol (13.902%), and *β*-pinene (11.230%), while in TEO, the predominant phytochemicals were: *γ*-terpinene (68.415%) and *p*-thymol (24.721%). The results indicated that the tested EOs alone as well as in combination have allelopathic effect against investigated seeds, while the synergistic effect of TEO and SEO in terms of fungal growth was demonstrated at a level of 0.06%. Thyme and sage EOs exhibited in vitro anti-proliferative activity on two melanoma cell lines, namely A375 human melanoma and B164A5 mouse melanoma alone, as well as in combination. SEO was most effective in terms of decreasing the cell viability of murine and human melanoma cell lines when compared to TEO.

## 1. Introduction

Sage (*Salvia officinalis* L.) and thyme (*Thymus vulgaris* L.) are medicinal aromatic plants of the Lamiaceae family grown on a large scale in southern and eastern Europe. The importance given to the two species lies in the multifunctionality of the active principles of these plants, found in extracts and volatile oils. These considerations, correlated with the current trends in the replacement of synthesis products with natural active compounds, have generated multiple uses for both plants in medicine, food, agriculture, horticulture, and plant protection. First of all, the medicinal plants of the Lamiaceae family have been recognized since ancient times for their therapeutical properties. Sage is used as a natural remedy in treating and curing arterial hypertension, bowel, stomach and spinal cord disorders, respiratory tract inflammation, physical and mental fatigue, nervousness, skin ulceration, cough, bronchitis, dental abscess, and cellulitis [1]. Thyme has proven calming action on cough, possesses anthelmintic and antiseptic properties [2]. The previous studies have highlighted the antibacterial effect of sage essential oil (SEO) and thyme essential oil (TEO), tested on multiple gram positive and negative bacteria [3,4,5,6,7,8,9,10]. In the recent years, a particular attention has been paid to the in vitro antitumor and antimutagenic properties of SEO and TEO. The antiproliferative effect of *Salvia* and *Thymus* species were tested on different human cancer cell lines [11,12,13,14,15,16,17,18].

Along with the therapeutic properties, an important role is represented by the possibility of using SEO and TEO as viable alternatives to synthetic products with herbicidal or antifungal activity. The antifungal properties of the analysed EOs and their constituents against *Aspergillus* sp., *Fusarium* sp. or *Penicillium* sp. have been demonstrated previously by several researchers [19,20,21,22,23,24,25,26]. Biological activity of EOs depends on their main chemotypes that are determined by the genotype and influenced by environmental and agronomic conditions [27]. In this regard, the main compound of TEO - thymol, is responsible for inhibiting the fungal growth and mycotoxins synthesis. Various mechanisms of action for volatile oils on microbial cells have been proposed over time. Thus, the volatile oils can affect both the outer coating of the cell and the cytoplasm. Their hydrophobic character appears to be responsible for disturbing the bacterial structures that lead to the increasing of the cytoplasmic membrane permeability [27].

Allelopathy is defined as the ability of a plant to produce biomolecules, especially secondary metabolites that may affect another plant beneficially, or vice versa [28]. The biochemical interactions between plants can have many practical applications in agriculture. Certain allelochimic compounds have been shown to be effective as herbicides. EOs of aromatic plants are explored to find out possible herbicides, since they do not persist in soil or contaminate the ground water and cause little or no mammalian toxicity [29]. The inhibition ability of EOs belonging to Lamiaceae family (lavender and peppermint) against weed germination (*Amarantus retroflexus* L., *Sinapis arvensis* L., *Lolium perenne* L.) has been previously reported. The results revealed the bio-herbicidal potential of EOs and the possibility to use them as natural herbicides [30].

The antifungal, antibacterial, antioxidant, and antitumoral activity exhibited by TEO and SEO have been demonstrated, but there are only few data concerning the synergism or antagonism of these EOs used together in different research areas. It seems that the use of mixed EOs is very beneficial in the field of medicine [31]. Also, to our best knowledge, there are limited studies on allelopathic effects of SEO and TEO.

This study is intended to be an innovative approach in terms of SEO and TEO applicability as natural functional products in agriculture and medicine. Thus, this multipurpose work has taken into consideration the following issues: (i) GC/MS characterization of SEO and TEO; (ii) evaluation of cytotoxic and antiproliferative activities of SEO and TEO against A375 human melanoma and B164A5 mouse melanoma cell lines; (iii) in vitro assessment of antifungal effect of both individual (SEO, TEO) and mixed (EOsM) against *Fusarium graminearum*; and, (iv) investigation on allelopathic potential of SEO and TEO in relation to crops and weeds seeds germination.

## 2. Results and Discussions

### 2.1. Chemical Composition of SEO and TEO

In Table 1 and Table 2 are presented the results regarding the chemical composition of analysed EOs and their liniar retention indices (LRI).

In SEO, there have been identified 14 major compounds, in a concentration over 0.2%. The main compounds identified in this essential oil, at a level over 10%, were: caryophyllene (25.364%), camphene (14.139%), eucalyptol (13.902%), and *β*-pinene (11.230%), representing 64.635% from all analysed components, Table 1. Also, other compounds as thymol (8.073%), camphor (4.028%), and valancene (5.525%) have been found in a proportion over 4%. The analysed compounds are divided in monoterpene hydrocarbonates (37.387%), sesquiterpene hydrocarbonates (25.364%), and monoterpene oxigenated (34.874%).

Our previous study has identified the presence of camphor (20.4%), eucalyptol (11.7%), camphene (11.5%), *α*-pinene (9.5%) as major compounds of the SEO that has been isolated from sage originating from western Romania [26]. Camphor has been reported as the main compound in different other studies [31,32], while other authors have identified *α*-thujone as main chemical compound [32,33,34]. The major similarity between the studies around the world regarding the SEO composition is represented by the fact that α-thujone was identified as the major compound, followed by camphor in a percentage of 20–25% of the total amount of chemical compounds.

In TEO, the *γ*-terpinene and thymol were identified as the major compound in proportion of 68.415% and 24.721% respectively. These results are in agreement with those previously published by other authors, who reported that the major component of thyme essential oil is thymol, a monoterpene phenol derivative of cymene [32,33,34]. Rus et al. [35] have reported that *o*-cymene and *γ*-terpinene are the main chemotypes of TEO. Regarding the type of compounds, monoterpene hydrocarbonates (MH) represent 68.415%, monoterpene oxigenated (MO) 25.299%, and sesquiterpene hydrocarbonates (SH) 6.063%.

The content of volatile compounds and the dominant chemotype varies with botanical specie, geographical origin, and the harvest time. Therefore, the study carried out by Imelouane et al. [36] have revealed camphor as the major compound (39.390%) of *Thymus vulgaris* L. essential oil cultivated in Morocco, while Hudaib et al. [33] have identified carvacrol, as the main chemotype of this essential oil. In another study conducted by Schmidt et al. [32], linalool has been found as the major compound of *Thymus vulgaris* L. essential oil (72.5%).

### 2.2. Cytotoxic Effect

**Figure 1:**
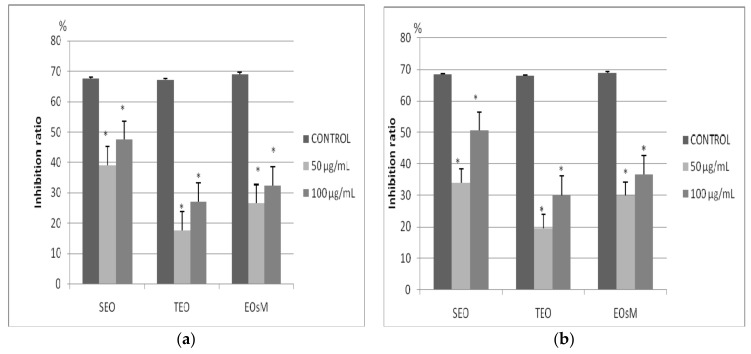
Antiproliferative activity of *Salvia officinalis* essential oil (SEO), *Thymus vulgaris* essential oil (TEO) and their essential oils mixture (EOsM) (**a**) for A375 human melanoma cell line; (**b**) for B164A5 mouse melanoma cell line: * the value is significant different versus control at *p* < 0.05.

Among the two volatile oils, SEO was more active in terms of inhibition of proliferation for the two tested melanoma cell lines. The inhibitory activity was linear with the concentration and quite similar for A375 human melanoma and B164A5 mouse melanoma cell lines. The lowest tested concentration of SEO (50 μg/mL) caused an inhibition ratio of 34% for the murine melanoma cell line and 39% for the human melanoma cell line, while a tested concentration of 100 µg/mL SEO lead to an inhibition ratio of 50.5% for the murine melanoma cell line and 47.5% for the human melanoma cell line. TEO at a tested concentration of 50 µg/mL induced an inhibition ratio of 19.5% for B164A5 cell line and 17.5% for A375 cell line, while a concentration of 100 µg/mL resulted in an inhibition ratio of 30% for B164A5 cell line and 27% for A375 cell line, Figure 1. Regarding the essential oils mixture (EOsM), the use of a concentration of 50 µg/mL conducted to a percentage of inhibition of 30% for B164A5 cell and 26.5% for A375 cells. The antiproliferative effect increases with the concentration of EOs.

In vitro antiproliferative activity against M14 human melanoma cells was reported for the essential oil obtained from other species of sage originating from Lebanon namely *Salvia bracteata* Banks & Sol and *Salvia rubifolia* Boiss [11]. In a comprehensive study, Russo et al. [15] have analyzed the chemical composition, and the growth-inhibitory and pro-apoptotic properties against A375, M14, and A2058 human melanoma cell lines of the SEO grown in eighteen different environmental conditions. The species showed in a different manner antiproliferative and pro-apoptotic properties on the selected melanoma cell lines, thus, demonstrating that the environmental and pedoclimatic conditions have a vital role on the chemical composition of volatile oil, and also, on the biological activity. *α*- and *β*-thujone isomers where assigned as responsible for the anti-melanoma activity, acting synergic with the other compounds present in the volatile oil [15].

It has been proven that the EO of *Salvia libanotica* showed in vivo chemoprevetive properties against skin cancer, experimentally induced with 7.12 dimethylbenz[*a*]anthracene (DMBA) and 12-0-tetradecanoylphorbol-13-acetate (TPA) [37].

The study conducted by Privitera et al. [38] investigated the antiproliferative activity of SEO at a level of 200 µg∙mL^−1^ for 72 h of incubation on two human lung cancer cell lines (A549 and NCI-H226), and significantly inhibition has been detected [38].

Together with the volatile oil obtained from other aromatic plants, the antiproliferative activity of the volatile oil of *Salvia triloba* L. was studied for MCF7 human breast adenocarcinoma cells. Interestingly, the ethanol extracts presented antiproliferative capacity but the aqueous extracts and the pure volatile oil, not [13]. In a broad study, analyzing the effect of the SEO, Loizzo et al. [39], have concluded that the cytotoxic effects were remarked for C32 amelanotic melanoma cell line, with an IC50 of 367 μg/mL and for ACHN renal adenocarcinoma human cell lines, with an IC50 of 108 μg∙mL^−1^. On the other hand, no effects were reported for LNCaP prostate carcinoma cell lines and MCF-7 human breast cancer cells. Evaluating the anticancer effects of *Salvia officinalis* L. volatile oil against UMSCC1, the squamous human cell carcinoma cell line of the oral cavity have described a dual effect, including both stimulation and inhibition, depending on the concentration. IC50 value has been reported to be 135 μg∙mL^−1^ [40]. Itani et al. [41] have noticed that three major components of *Salvia libanotica* EO, namely linalyl acetate, terpeniol, and camphor caused growth inhibition and apoptosis for human colon cancer HCT116 cells both p53^+/+^ and p53^−/−^.

The volatile oil of thymus endemic species from Algeria and Morocco was found to show cytotoxic properties against A375 human melanoma cell line with an IC50 46.95 μg·mL^−1^ [42]. A recent study depicted the antiproliferative activity against THP-1 human monocytic cell line of the volatile oil obtained from different species of thymus originating from Portugal [43]. The findings were supported by the group of Tefiani et al. [44] that concluded that *Thymus munbyanus* volatile oil collected from Algeria present remarkable antiproliferative effects against THP-1 human acute monocytic leukemia cell line.

The essential oils of two thymus species, namely *Thymus linearis* Benths., and *Thymus serpyllum* L. from the Pakistan flora, have been screened for antiproliferative effects against two human cancer (MCF-7-breast and LNCaP-prostate carcinoma) and one fibroblast (NIH-3T3) cell lines. Both volatile oils showed antiproliferative potential on the above mentioned cell lines, but the effectiveness of the volatile oil obtained from *Thymus linearis* Benths. has been significantly increased with IC50 of 80.7 μg·mL^−1^, 70.3 μg·mL^−1^ and 100.2 μg·mL^−1^ [45]. A recent study has shown the cytotoxic effect of *Thymus caramanicus* Jalas, a common species in Iran, against KB human oral epidermoid carcinoma [46].

### 2.3. Antifungal Activity

In Table 3, the results regarding the antifungal activity of SEO and TEO are shown on *Fusarium graminearum* mycelium growth area (MGA). At a closer look, it can be noted that the highest radial grow increase of mycelium was recorded in sample treated with SEO at level of 0.06%, where the MGA value was about 15.9 cm^2^. By applying of SEO and TEO mixture at a level of 0.06% (in the same proportion) was obtained only 0.1 cm^2^ value of MGA. This finding reveals the synergistic effect of SEO and TEO. Similarly, the previous studies on this topic have shown that the antifungal effect is not only caused by a major compound as by the synergy of the other compounds found in smaller amounts [47,48].

With respect to the antifungal potential of TEO, our results revealed a total inhibition of *Fusarium* mycelium growth at all investigated concentrations. Thus, the mycelia radial growth (RG) in sample with TEO was similar to thiophanate methyl (as negative control), the MGA values being null (in Table 3).

Although by applying the EOsM at 0.06% an increase of mycelium was recorded, the differences are not statistically significant (*p* > 0.05) versus negative control. The antifungal effect of sage oil has been highlighted on other fungal species. Mahmoudi et al. [49] have investigated the effect of SEO on *Alternaria alternata*, and reported that a level of 5 mg/L was the minimum inhibitory concentration against the fungus growth. The same effect has been shown for *Botritis cinerea* and *Fusarium* sp. [50,51]. Additionally, it has been proved that the SEO showed antifungal activity against dermatophyte strains [52].

The antifungal potential of TEO has been demonstrated also on other types of fungi. Thus, the study performed by Tantaoui-Elaraki et al. [53] investigated the effect of TEO on *Aspergillus parasiticus* in vitro conditions and reported that the minimum inhibitory concentration for this type of fungi was 0.1%. Also, it has been proved that TEO has shown a strong antifungal activity on *Verticillium dahliae*, *Fusarium* sp., *Penicillium* sp., and *Aspergillius* sp. [54]. Among *Thymus*, *Rosmarinus*, and *Eucalyptus*, essential oils it has been found that TEO showed the lowest inhibitory concentration against the fungal growth [55].

### 2.4. Allelopatic Potential

**Figure 2:**
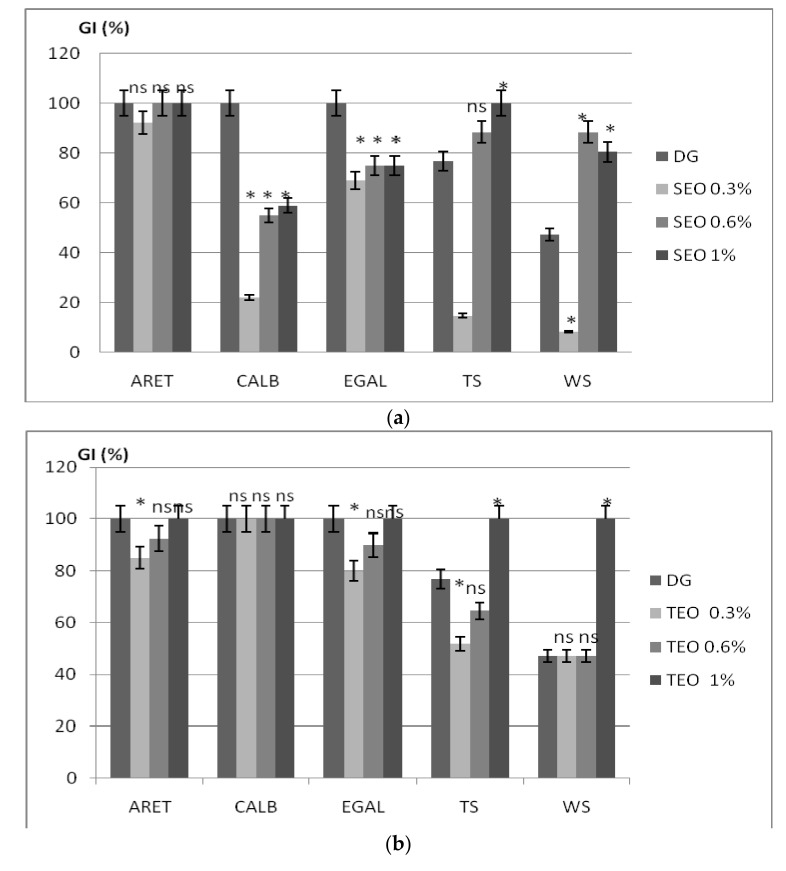

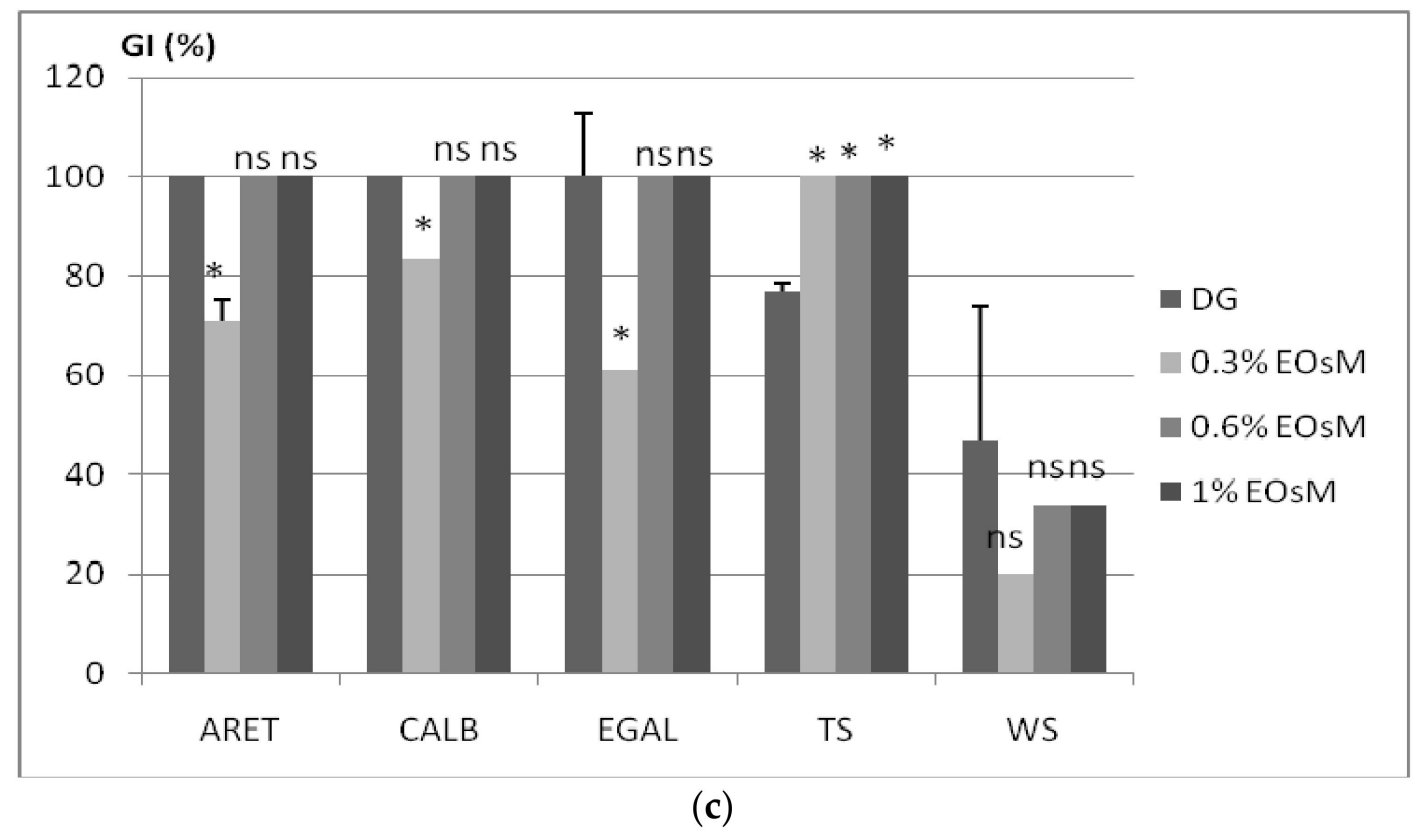
The effect of (**a**) *Salvia officinalis* essential oil—SEO; (**b**) *Thymus vulgaris* essential oil—TEO; and (**c**) their mixtures (EOsM) on seeds germination rate (GI) of *Amaranthus retroflexus* (ARET), *Chenopodium album* (CALB), *Echinochloa crus-galli* (EGAL), tomato (TS), and wheat (WS). * the value is significant different versus the control with Dual Gold (DG) at *p* < 0.05; ns—the value is no significant versus control.

The results regarding the herbicidal effect of the investigated EOs compared to positive control as synthetic herbicide Dual Gold (960 g L^−1^ s-metolachlor), (DG) are presented in the Figure 2. The DG completely inhibits the weeds germination, while the tomato seeds are inhibited in percentage of 76.8% and wheat seeds in proportion of 47.2%.

Our results have proven that SEO showed herbicidal effect on *Amaranthus retroflexus* L. (ARET), even when it was used at low concentrations. Thus, when SEO was applied at a level of 0.3%, the inhibition of germination was 92.1%, while by increasing the level of SEO to 0.6% or 1.0%, total inhibition of germination of ARET seeds occurred. Controlling the germination of *Chenopodium album* L. (CALB) weeds by using Dual Gold is 100% effective at recommended dose of 1 L per hectare. The same value of germination inhibition rate (GI) of CALB weed is also achieved by spraying the seeds with SEO at a level of 1.0%. The herbicidal capacity of SEO decreases with the dose. Thus, by applying SEO at a level of 0.6% resulted of 55.5% value of GI, while a dose of 0.3% SEO led to 22.2% value GI.

The herbicidal effect of SEO on *Echinochloa crus-galli* L. seeds (EGAL) has reached a maximum value of GI about 75% by applying SEO at a level of 0.6% and 1.0%, while a level of 0.3% SEO induced the lowest herbicidal effect of 69.1%.

The study of the allelopathic effect of EOs on wheat and tomato seeds shows that the SEO, applied at a level of 0.6% resulted in a GI value of 88.4% while a level of 1.0% has the ability to inhibit the germination in a percentage of 100% for tomato seeds and 80.5% for wheat seeds.

Using SEO al a level of 0.3% it was noted a decrease in the germination inhibition of tomato seeds up to 14.8%, respectively up to 8.3% in wheat seeds, as compared to the synthetic herbicide that showed a better ability to inhibit the seeds germination (76.8% for tomatoes and 47.2% for wheat). Thus, there are no significant differences versus DG as positive control by using SEO as an herbicidal agent for ARET, CALB seeds (except 0.6% SEO when the statistical differences were significant, *p* < 0.05) and EGAL (except 1.0% SEO when the statistical differences were significant, *p* < 0.05). SEO applied at a level of 1.0% induced significant difference (*p* < 0.05) versus positive control in GI of tomato seeds, while no significant differences versus positive control were recorded in the GI of wheat seeds.

By applying TEO at different doses, only for a level of 0.3% there were recorded significant differences (*p* < 0.05) in the GI of ARET, EGAL, and tomatoes versus DG as a positive control.

As regards the essential oils mixtures (EOsM), when was applying at a level of 0.3% in the same proportion, significant differences were recorded in the GI of ARET versus positive control. Also, the using of 0.6% EOsM induced significant differences in the GI value of EGAL versus the positive control.

All of the investigated levels of EOsM induced no significant differences in GI of wheat seeds versus positive control, while significant differences were noted in the GI of tomato seeds versus positive control.

The ability of SEO at a level of 0.3% to inhibit the germination of weed seeds and to slightly affect the germination of wheat and tomato seeds could recommend the use of this essential oil in plant protection.

Our results have proven that the TEO shows better herbicidal potential than SEO. Thus, at TEO applied at a level of 0.6% and 1.0% has completely inhibited the germination of CALB and EGAL seeds and in a proportion of 60.6% in the case of ARET seeds.

By decreasing the level of TEO at 0.3% do not record a great inhibition of the seeds germination of ARET (6.6%) and CALB (10%). Unfortunately, TEO showed an inhibition effect on organic tomato and wheat seeds, at over 80%, even used at low doses.

The synergistic effect of the investigated oils resulted in increasing the allelopathic potential of their mixture and in a total inhibition of germination on all types of weeds, when EOsM was applied at a level at least 0.6%. It is worth noting that the use of the mixture does not inhibit the wheat seeds germination, thus recommending the blending of the two EOs in equal proportion, as bio-effective herbicides in the control of weed grain crops.

When compared to synthetic herbicide DG, the investigated EOs mixtures exert an increased efficiency without having a negative effect on the wheat germination. However, the synergistic effect of the two EOs, even applied at a low level, leads to a complete inhibition of tomato seeds germination.

The inhibitory capacity of EOs applied both individually and as a mixture, can be strongly influenced by their chemical composition and also by the generated synergism. It has been reported that the terpenoids, particularly the sesquiterpene, even at a low level, exhibited specific structure-activity relationship, which can generate phytotoxicity [56].

The previous studies have highlighted the bio-herbicidal effects of EOs belonging to Lamiaceae family (oregano and rosemary) on weeds control and germination growth of bread wheat cultivars [31]. The results have shown that 2–4 μL of EOs exhibits an inhibition effect on *Sinapis arvensis* L., but not against *Avena sterilis* L. seeds. Moreover, these seeds have survived at all doses of oregano and rosemary EOs. Other studies [57] revealed the inhibition potential of peppermint, cinnamon, and lavender oils against redroot pigweed, while ryegrass and wild mustard seeds germination. When compared with the control, peppermint oil has been the more effective in inhibiting ryegrass seed germination. Study regarding the effect of eucalyptus, camphor, and lemongrass EOs on seed germination and seedling growth of *Parthenium hysterophorus* shown that a higher level than 12 mL/L induced a total inhibition of weed germination [56].

Our results revealed significant differences in the inhibitory effect of the investigated EOs on germination capacity of wheat and tomato versus weed species. These findings could be assigned to the different rates to metabolize certain monoterpenes [31]. Thus, the volatile compounds may also be responsible for the inhibition of seed germination [58]. Wheat and tomato seeds were less affected than the weed seeds species, which highlights that the EOs, applied in a proper dose could be recommended as a bio herbicide for weed control.

## 3. Materials and methods

### 3.1. Plant Material

Aerial parts of sage and thyme were collected in July and August of 2015 from the experimental field of Banat’s University of Agricultural Sciences and Veterinary Medicine “King Michael I of Romania” from Timisoara at the full flowering stage. Voucher specimens were deposited in the Herbarium of Medicinal Plants Department of Agriculture Faculty. The plants were dried at ambient temperature and were ground with the Grindomix Retsch GM 2000 laboratory mill. Weed seeds of *Amaranthus retroflexus* (ARET), *Chenopodium album* (CALB), *Echinochloa crus-galli* (EGAL) used in allelopathic test were obtained from the Spontaneous Plant Seeds Collection of the Department of Herbology from Faculty of Agriculture Timisoara. Tomato seeds (TS), Saint-Pierre variety, and wheat seeds (WS) Lovrin variety were purchased from local market.

### 3.2. Extraction of Essential Oils

SEO and TEO were obtained from dried plants by hydrodistillation using a Clevenger equipment. 300 g of plant material was introduced into the apparatus with 3000 mL tap water. The mixture was heating to the evaporation temperature of the oil. The extraction time is considered after the complete distillation of the oil and the recovered of another 500 mL of flavored water. The obtained EOs was kept at 2–4 °C in dark conditions until their using for in vitro and in vivo experiments.

### 3.3. GC-MS Analysis

Agilent Technology 7820A (AGILENT Scientific, Santa Clara, CA, USA), coupled with mass spectrometer MSD 5975 and equipped with a capillary column DB WAX (30 m × 250 μm × 0.25 μm) was used. The carrier gas was helium with a mass flow of 1 mL/min. In order to separate the compounds, the following GC oven program was used: 40 °C for 1 min, 5 °C min^−1^ to 210 °C for 5 min. The injector and ion source temperatures were 250 and 150 °C, respectively. The injection volume was 1 μL of each pure oil or mixture, without solvent with a split ratio 1:20. One run for each sample was performed. The NIST spectra library has been used to identify the volatile compounds. Identification was made by comparison of their mass spectra with those stored in NIST 02, Wiley 275 libraries. The percentages of individual components were calculated based on GC peak areas without using correction factors. The linear retention indices (LRI) were determined in relation to a homologous series of *n*-alkanes (C8–C24) under the same operating conditions and calculated according to Van den Dool and Kratz [59] Formula (1):

(1) $$\mathrm{LRI}=100\times n+\frac{100\times (t_{x}-t_{x})}{t_{n+1}-t_{n}}$$

where t_n_ and t_n+1_ are the retention times of the reference *n*-alkane hydrocarbons eluting immediately before and after chemical compound “X” and t_x_ is the retention time of compound “X”.

### 3.4. MTT Proliferation Assay

The inhibition of proliferation for the screened extracts was assessed for A375 human melanoma and B164A5 mouse melanoma cell lines. The above-mentioned cell lines where acquired from Sigma-Aldrich Company, Ayrshire, UK. Cells were cultured in complete growth medium containing Dulbecco’s Modified Eagle’s Medium (DMEM; Gibco BRL, Invitrogen, Carlsbad, CA, USA), supplemented with 10% fetal calf serum (FCS; PromoCell, Heidelberg, Germany), 1.0% Penicillin/Streptomycin mixture (Pen/Strep, 10,000 IU∙mL^−1^, FCS; PromoCell), and 2% HEPES (4-(2-hydroxyethyl)-1-piperazineethanesulfonic acid; Gibco BRL). Antiproliferative activity was assessed by MTT assay. On this purpose, the two melanoma cell lines were seeded onto a 96-well culture plate at a cellular density of 6000 cells per well and were attached to the bottom of the well overnight. After 24 h, 100 μL of new medium containing Dulbecco’s Modified Eagle’s Medium (DMEM; Gibco BRL, Invitrogen, Carlsbad, CA, USA) and 50 µg∙mL^−1^ respective 100 µg∙mL^−1^ of the tested samples (dissolved in dimethyl sulfoxide—DMSO; Sigma-Aldrich Company) were added and incubated for 72 h. The melanoma cells were then assayed by the addition of 10 μL of 5 mg∙mL^−1^ MTT (3-(4,5-Dimethylthiazol-2-yl)-2,5-diphenyltetrazolium bromide) solution from the MTT-based in vitro toxicology assay kit (Tox-1; Sigma-Aldrich Company). The intact mitochondrial reductase converted and precipitated MTT as blue crystals during a 4 h contact period. The precipitated crystals were dissolved in 100 μL of lysis solution provided by the manufacturer (Sigma-Aldrich Company). Finally, the reduced MTT was spectrophotometrically analyzed at 570 nm, using a microplate reader Bio-Rad Mark Microplate Spectrophotometer).

### 3.5. Antifungal Activity

The in vitro antifungal potential of SEO and TEO has been evaluated on CYGA medium (cloraphenicol-yeast-glucose–agar) (Sigma Aldrich Chemie, Madrid, Spain) for mycelium radial growth (RG) of *Fusarium graminearum* (Fg 06_17). The fungus was acquired from Microbial Collection of Microbiology Department, Faculty of Horticulture, and Forestry. The method used was “poisoned food technique” which involves the introduction into the culture media of different amounts of essential oil [60]. Thus, in our study, SEO and TEO were individually applied at three concentrations of 0.06%, 0.12% and 0.2%. To investigate the synergistic effect of the two investigated oils, the essential oils mixture (EOsM) in equal proportion (*v*/*v*) has been included al the same concentrations of 0.06%, 0.12%, and, respectively, 0.2%. Also, a negative control (C) with a commercial fungicide was used as thiophanate methyl at a level of 0.1%. The *Fusarium* mycelium as inoculums was get from a four days young mycelium obtained from single spore technique, [61]. Plugs, with 8 mm diameter were harvested from edge of mycelium and transferred on CYGA media added with EOs. Three plugs for each variant were used as replicates. The Petri dishes at 24 ± 30 °C in reverse position and a photoperiod of 12 h/day were incubated. In the 5th day, was recorded the mycelium radial grow value (RG) expressed in mm, calculated as arithmetic mean of the two perpendicular diameters, using a ruler. The mycelium growth area (MGA) was estimated using the formula:

(2) $$\mathrm{MGA}(cm^{2})=\frac{\frac{RG^{2}\times 3.14}{4}-50.24}{100}$$

where the value of 50.24 mm^2^ represents the area of fungal plug (inoculum), calculated on the base of fungal plug diameter (8 mm).

For EO concentrations where no fungal growth was recorded, the confirmation test was performed. This involved the transfer of fungal plugs on fresh CYGA without EOs addition. In the case that after four days there was not recorded any fungal growth, the EO concentration from which the transfer has been performed is considered to have a fungistatic effect. Contrary, if it was noticed any fungus revival, the EO concentration from the medium on which the transfer was made is considered to be fungicidal.

### 3.6. Allelopatic Potential

Seeds were sterilized by using sodium hypochlorite solution (1:9), and washed two times with distilled water. A total of 10 seeds from each plant were placed in a Petri dish. Then, the seeds were treated with each essential oil at a level of 0.3%, 0.6% and 1.0%. The dilutions of EOs were done with distillated water. To point out the synergism of the investigated oils, the EOsM in equal proportion (*v*/*v*) has been tested at a level of 0.3%, 0.6% and 1.0%. Two control samples have been carried out, as follows: a negative control with water and a positive control with 0.003% Dual Gold 960 EC (s-metolachlor) by SYNGENTA (GD). The Petri dishes were transferred to a growth chamber and kept at 25 °C into the dark. The number of germinated seeds in each Petri dish was recorded after seven days. A seed was considered germinated when the radicles was elongated up to 3 mm. The germination percentage was determinate using the Equation (3) [62]:

(3) $$\mathrm{GI}(\%)=\frac{\mathrm{GC}-\mathrm{TG}}{\mathrm{GC}}\times 100$$

where GI represents the rate of seeds germination inhibition, GC is germinated seeds in negative control (with water), and TG represents germinated seeds in the samples treated with EOs.

### 3.7. Statistics

The results of this study were presented as mean ± standard deviation (SD). All of the in vitro experiments for assessing the anti-proliferative potential of EOs and their mixtures have been performed on two micro plates in quadruplicates for each tested substance. Student’s T-Test (Microsoft Excel 2007) was applied to evaluate the statistical significance of differences recorded within the data regarding the allelopatic potential EOs and their mixtures. Each group was individually compared with the positive control and the statistical differences were shown, as follows: *: significant (*p* < 0.05); **: highly significant (*p* < 0.01); ***: extremely significant (*p* < 0.001); and, ns: non-significant (*p* > 0.05). Also, the statistical analysis of the results concerning the antifungal activity has been performed on the base of Student’s *T*-Test.

## 4. Conclusions

The results of present study confirmed the allelopathic activity of sage and thymus essential oils and the ability to inhibit the germination of investigated weed species. The synergism of these oils induced the increasing of bio-herbicidal effect revealed at lower concentrations. The mixtures of oils could be recommended for *Amaranthus retroflexus*, *Chenopodium album*, and *Echinochloa crus-galli* control in wheat fields.

The antifungal potential of thymus essential oil demonstrated a total inhibition of *Fusarium* mycelium growth at all investigated concentrations and a synergistic antagonistic effect with sage essential oil highlighted at low concentration.

Among the two volatile oils, sage essential oil was more active in terms of inhibition of proliferation of A375 human melanoma and B164A5 mouse melanoma cell lines.

When considering all of these aspects it is recommended to use the mixture of investigated essential oils in the purpose of potentiating the antifungal, herbicidal, and anti-proliferative effects in organic farming and medical practice.

## Figures and Tables

**Table 1:** Chemical composition of *Salvia officinalis* essential oils (SEO).

No. Crt.	Compounds	Type of Compound	Linear Retention Indices (LRI)	% of Total
**1**	*α*-pinene	MH ^1^	1013	4.816
**2**	*β*-pinene	MH	1092	11.230
**3**	eucalyptol	MO ^2^	1198	13.902
**4**	*o*-cymene	MH	1287	0.515
**5**	*α*-thujone	MO	1416	8.871
**6**	camphor	MO	1493	4.028
**7**	camphene	MH	1498	14.139
**8**	d-carvone	MO	1518	0.205
**9**	*m*-menthane	MH	1573	0.853
**10**	*p*-thymol	MO	1578	8.073
**11**	caryophyllene	SH ^3^	1581	25.364
**12**	*trans*-calamenene	MH	1644	1.676
**13**	valencene	MH	1713	5.525
**14**	terpinolene	MH	1774	0.360
Total				97.624
		MH		37.387
		SH		25.364
		MO		34.874
		% of SEO extraction		3.560

^1^ MH-Monoterpene hydrocarbonates; ^2^ MO-Monoterpene oxygenated; ^3^ SH-Sesquiterpene hydrocarbonates.

**Table 2:** Chemical composition of *Thymus vulgaris* essential oils (TEO).

No. crt.	Compounds	Type of Compound	Linear Retention Indices (LRI)	% of Total
**1**	*γ*-terpinene	MH ^1^	1250	68.415
**2**	*p*-thymol	MO ^2^	1578	24.721
**3**	Caryophyllene	SH ^3^	1581	5.508
**4**	Terpine-4-ol	MO	1635	0.099
**5**	*o*-thymol	MO	1745	0.237
**6**	*p*-cymene-7-ol	MO	1824	0.242
**7**	Caryophyllene oxide	SO ^4^	2013	0.222
**8**	*α*-murolene	SH	2120	0.555
Total				98.644
		MH		68.415
		SH		6.063
		MO		25.299
		SO		0.222
		% of TEO extraction		3.040

^1^ MH-Monoterpene hydrocarbonates; ^2^ MO-Monoterpene oxygenated; ^3^ SH-Sesquiterpene hydrocarbonates; ^4^ SO-Sesquiterpene oxygenated.

**Table 3:** Antifungal activity of SEO and TEO on *Fusarium graminearum* mycelium growth.

Variants	*Fusarium graminearum*	
	^1^ RG (mm)	^2^ MGA (cm^2^)
Control	46.8 ^a^ ± 2.4	16.7 ^a^ ± 1.8
0.06% SEO	45.8 ^b^ ± 2.5	15.9 ^b^ ± 1.7
0.12% SEO	32.8 ^c^ ± 2.0	7.9 ^c^ ± 1.0
0.20% SEO	20.7 ^d^ ± 1.8	2.8 ^d^ ± 0.6
0.06% TEO	8.0 ^d^ ± 0	0.0 ^e^
0.12% TEO	8.0 ^d^ ± 0	0.0 ^e^
0.2% TEO	8.0 ^d^ ± 0	0.0 ^e^
0.06% EOsM	9.0 ^d^ ± 0	0.1 ^e^ ± 0
0.12% EOsM	8.0 ^d^ ± 0	0.0 ^e^
0.2% EOsM	8.0 ^d^ ± 0	0.0 ^e^
0.1% thiophanate methyl	8.0 ^d^ ± 0	0.0 ^e^

^1^ Radial Grow; ^2^ Mycelium Growth Area; Within a column the values with different superscript letters are significantly different (*p* ˂ 0.05).
